# Impact of explainable artificial intelligence assistance on clinical decision-making of novice dental clinicians

**DOI:** 10.1093/jamiaopen/ooac031

**Published:** 2022-05-17

**Authors:** Aaron Glick, Mackenzie Clayton, Nikola Angelov, Jennifer Chang

**Affiliations:** 1 General Practice and Dental Public Health, University of Texas Health Science Center at Houston School of Dentistry, Houston, Texas, USA; 2 Primary Care and Clinical Medicine, Sam Houston State University College of Osteopathic Medicine, Conroe, Texas, USA; 3 University of Texas Health Science Center at Houston School of Dentistry, Houston, Texas, USA; 4 Periodontics and Dental Hygiene, University of Texas Health Science Center at Houston School of Dentistry, Houston, Texas, USA

**Keywords:** artificial intelligence, clinical decision support systems, furcation defect, radiography, dental, decision-making

## Abstract

**Objective:**

Despite artificial intelligence (AI) being used increasingly in healthcare, implementation challenges exist leading to potential biases during the clinical decision process of the practitioner. The interaction of AI with novice clinicians was investigated through an identification task, an important component of diagnosis, in dental radiography. The study evaluated the performance, efficiency, and confidence level of dental students on radiographic identification of furcation involvement (FI), with and without AI assistance.

**Materials and Methods:**

Twenty-two third- and 19 fourth-year dental students (DS3 and DS4, respectively) completed remotely administered surveys to identify FI lesions on a series of dental radiographs. The control group received radiographs without AI assistance while the test group received the same radiographs and AI-labeled radiographs. Data were appropriately analyzed using the Chi-square, Fischer’s exact, analysis of variance, or Kruskal–Wallis tests.

**Results:**

Performance between groups with and without AI assistance was not statistically significant except for 1 question where tendency was to err with AI-generated answer (*P* < .05). The efficiency of task completion and confidence levels was not statistically significant between groups. However, both groups with and without AI assistance believed the use of AI would improve the clinical decision-making.

**Discussion:**

Dental students detecting FI in radiographs with AI assistance had a tendency towards over-reliance on AI.

**Conclusion:**

AI input impacts clinical decision-making, which might be particularly exaggerated in novice clinicians. As it is integrated into routine clinical practice, caution must be taken to prevent overreliance on AI-generated information.

## Lay Summary

Artificial intelligence (AI) is being used with increasing frequency in the healthcare field to provide earlier and easier detection of abnormalities. Although these AI systems are designed to optimize accuracy in detecting abnormalities less is known about the interaction of the clinician and system. We tested the interaction of an AI system with novice clinicians (dental students) that were attempting to diagnose abnormalities in dental radiographs. One group of the dental student participants received AI assistance, whereas the other group did not receive AI assistance. We investigated 3 primary metrics during AI and participant interactions: (1) performance, (2) efficiency, and (3) confidence. Our findings suggest that novice clinicians are more likely to over-rely on AI leading to potential lower performance when assisted with an AI system. In addition, the AI system used in this study did not improve decision-making speed or confidence in novice clinicians. Despite the limitations of this single study, those that are developing AI systems to aid in clinician decision-making should keep in mind the psychological interaction (machine/human) and end user experience that can potentially affect clinical performance and patient safety. Additionally, participants that used AI and did not use AI assistance felt that these systems have the potential to improve clinical decision-making.

## BACKGROUND AND SIGNIGICANCE

Artificial intelligence (AI) is increasingly being used in the healthcare field to provide clinicians with evidence-based decision aids.^1^ In the field of dental radiology, AI programs have been used to identify radiographic landmarks in orthodontic treatment planning, diagnose maxillofacial cysts and tumors, classify lesions associated with teeth, estimate the degree of alveolar bone loss, and detect dental caries among other applications.^2^

Ideally, the use of AI increases the efficiency and accuracy of clinical decision-making, leading to better patient outcomes.^3^ However, while accuracies of AI models are often reported the accuracies during implementation clinically as a decision support system are rarely assessed. Further, despite the accuracy of AI models utilized in the identification of medical/dental conditions implementation of these systems pose potential biases and risks in clinical diagnosis. In particular, confirmation bias and the anchoring effect can affect diagnostic accuracy.^4^ If a clinician suspects a particular diagnosis and an AI program agrees, the clinician might fail to consider other possible diagnoses. Similarly, a clinician could become anchored to the diagnosis suggested by an AI program and only consider it moving forward.^5^^,^^6^ Automation bias and complacency can also affect clinicians using AI programs.^6^ Automation bias would be underestimating the AI error and thus accept AI-generated input as ground truth. An example of complacency bias would be where a clinician suspects the AI-generated input is incorrect yet declines to investigate further simply because it was computer generated. The aforementioned biases can interfere with the accuracy of a diagnosis, and clinicians might be more susceptible to these biases when presented with data from an AI program.^4^^,^^7^

As the accuracy of AI-based models continues to increase, the implementation of these systems as a clinical decision support system will become increasingly important. Most studies have been consistent in their findings that the use of AI programs increases efficiency and minimizes the amount of time clinicians spend on tasks.^8^^,^^9^ Therefore, AI has great potential to improve patient care, but susceptibility to potential biases must be minimized for its successful implementation in healthcare. Healthcare providers, especially those who are less experienced, can over-rely on the data provided by AI programs, leading to inaccurate diagnoses.^4^ Diagnostic sensitivity can even decrease among radiologists when using AI programs compared with when they do not.^10^ Thus, action is needed to reduce tunnel vision by healthcare providers and ensure that AI programs are truly enhancing clinical decision-making. Asan *et al.*^11^ recommend cultivating a healthy level of skepticism towards AI-generated information in order to limit over-reliance on computer programs while utilizing their benefits. They suggest that increasing fairness, transparency, and robustness of AI programs will help create the ideal, balanced relationship between healthcare providers and AI. AI programs will continue to be refined and improved, but it is imperative to develop the correct level of trust between AI and clinicians to maximize diagnostic accuracy during clinical implementation.

Radiographic interpretation plays an important role in diagnosis of periodontitis, a common inflammatory disease that causes bone loss and eventual tooth loss.^12^ Furcation involvement refers to the loss of alveolar bone between the roots of multirooted teeth and impacts the prognosis of the involved tooth.^13^^,^^14^ Due to dental student’s limited clinical experience, they often struggle with radiographic diagnosis of furcation involvement. This study evaluated the use of AI as a diagnostic tool for furcation involvement for dental students. A convolutional neural network (CNN) model with was designed to identify furcation involvement radiographically with an accuracy of 81% using labeling from calibrated periodontists as gold-standard. It was hypothesized that use of the CNN would increase the performance of radiographic furcation involvement identification by dental students.

## OBJECTIVE

The goal of this study was to evaluate the performance, confidence, and efficiency of dental students in the identification of radiographic furcation involvements with and without CNN assistance. Additionally, the study was aimed to evaluate student perception regarding the use of AI in clinical decision-making. It was hypothesized that students with CNN assistance would have increased (1) performance, (2) efficiency, and (3) confidence than students without CNN assistance, and that students would support the use of AI in healthcare.

## MATERIALS AND METHODS

### Study design and participants

Approval from the Committee for Protection of Human Subjects of University of Texas Health Science Center at Houston was obtained (HSB-DB-20-1358). Prior to subject recruitment, the U-Net: Convolutional Networks for Biomedical Image Segmentation^15^ was used to create the CNN model to identify radiographic furcation involvements. A particular benefit of this training network is upsampling that allows propagation of context information. Therefore, training data represented is larger than in other CNN models. The model used 4 shortcut connections to increase training efficiency. Typical training and testing datasets were used with a success rate of 81% to identify furcation involvement (FI). Two surveys using 3 deidentified radiographs with maxillary/mandibular molars with/without buccal/lingual furcation involvements were composed. Of which, 4 out of the 5 teeth had been labeled by the CNN as accurate as compared with calibrated periodontists. One survey included assistance of CNNs, one did not. Only dental students with clinical patient care experiences from the University of Texas School of Dentistry were included. Twenty-two third-year students (DS3) and 19 fourth-year students (DS4) who responded to the recruitment messages were randomly assigned equally into groups that completed surveys with and without CNN assistance.

### Questionnaire

**Figure 1.**
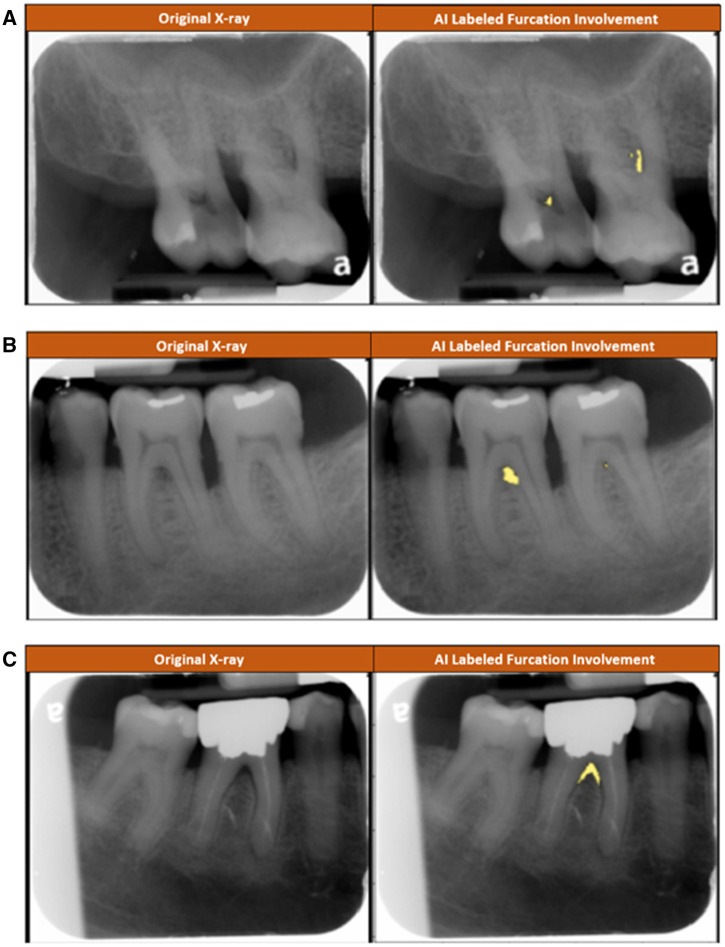
Images provided in the survey. The participants in the control group received the original radiograph only. The participants in the test group received the original radiograph and the AI-labeled radiograph. Yellow color appearing on AI-labeled radiographs denote identification of furcation involvement. (A) The participants were asked to identify if there was a furcation involvement in tooth no. 3, the molar on the right of the original image. (B) The participants were asked to identify if there was a furcation involvement in teeth nos. 18 and 19, the molars on the right and left of the original image, respectively. (C) The participants were asked to identify if there was a furcation involvement in teeth nos. 30 and 31, the molars on the right and left of the original image, respectively.

The survey (Table 1) consisted of 9 questions: year of training, general confidence level regarding radiographic identification of furcation lesions before the survey, 5 questions with maxillary/mandibular molar radiographs inquiring about the presence or absence of buccal or lingual furcation lesions (Figure 1), postsurvey question about confidence level regarding accuracy in identification of the lesions, and postsurvey question about the usefulness of a computer program for identifying furcation lesions radiographically. Confidence level was selected from the following Likert-scale questions: (1) very unconfident, (2) not confidence, (3) neutral, (4) confident, and (5) very confident. Participants were also asked their opinion on the usefulness of a computer program in identification, participants selected if it was (1) very unlikely, (2) not likely, (3) neutral, (4) likely, or (5) very likely to help improve clinical confidence.

**Table 1.** Questionnaire

1. What is your year of training?	DS3 DS4
2. Before we get started, what is your confidence level on radiographic identification of furcation lesions?	Very unconfident Not confident Neutral Confident Very confident
3. Please identify if there is a furcation lesion on tooth no. 3 (molar on the right of the original image). Figure 1A presented.	Yes No
4. Please identify if there is a furcation lesion on tooth no. 18 (molar on the right of the original image). Figure 1B presented.	Yes No
5. Please identify if there is a furcation lesion on tooth no. 19 (molar on the left of the original image). Figure 1B presented.	Yes No
6. Please identify if there is a furcation lesion on tooth no. 30 (molar on the right of the original image). Figure 1C presented.	Yes No
7. Please identify if there is a furcation lesion on tooth no. 31 (molar on the left of the original image). Figure 1C presented.	Yes No
8. What is your confidence level on your diagnostic accuracy of identifying previous radiographic lesions?	Very unconfident Not confident Neutral Confident Very confident
9. Do you think a computer program for practicing on radiographic furcation lesion identification would help your clinical confidence?	Very unlikely Not likely Neutral Likely Very likely

### Statistical analysis

Prior to data collection, a power analysis was performed resulting in a sample size 20 participants per group that would provide 80% power to detect a difference in the correct responses of 20% or less between groups. The results of the surveys were statistically analyzed using R statistical software.^16^ The chi-square and Fisher’s exact tests were used to identify differences in accuracy and confidence (presurvey confidence and postsurvey confidence) between the test and control group as well as a subgroup analysis within third- and fourth-year students. A 1-way analysis of variance (ANOVA) was used to detect change in confidence from pre- to postsurvey compared between test and control groups. ANOVA was also used to detect statistically significant differences in the amount of time spent on each question between and within the test and control groups. Significance level of *P* = .05 was used for all tests.

## RESULTS

A total of 21 students (12 third-year dental students and 9 fourth-year dental students) completed the control survey and 20 students (10 third-year dental students and 10 fourth-year dental students) completed the test survey.

**Figure 2.**
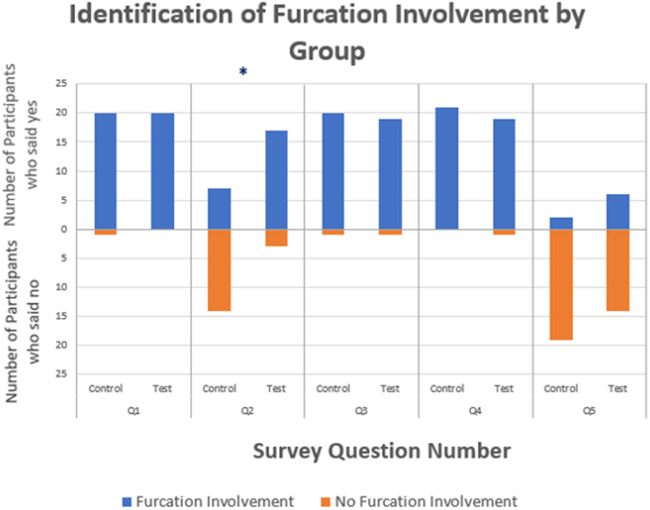
Graph of identification of furcation involvement per question by group. Number of study participants who reported presence or absence of furcation involvement in the radiographic figures presented (*indicates significance with *P* < .05).

There was no significant difference in agreement between the test and control groups regarding classification of furcation involvement for all questions except question 2 (*P *<* *0.05; Figure 2). In question 2, the control group was more likely to not identify a furcation involvement while the test group with CNN assistance was more likely to agree with CNN presented data of identifying a furcation involvement.

Participants without CNN assistance spent 91.59 ± 121.67 seconds identifying the 5 furcation involvement lesions, and participants with CNN assistance spent 70.98 ± 29.18 seconds identifying the 5 furcation involvement lesions. There was no statistically significant difference between groups. In a subgroup analysis, there was also no statistically significant difference in time spent identifying the 5 furcation involvement lesions between third- and fourth-year dental students (96.05 ± 32.19 and 64.73 ± 34.18, respectively). Time spent per question per group did not differ significantly (Table 2), yet in a subgroup analysis for question 2, question with most deviation in agreement between groups, the third-year dental students spent more time for this question than the fourth-year dental students in the group presented with CNN assistance (19.64 ± 3.30 and 11.34 ± 1.58, respectively; *P *=* *.02).

**Table 2.** Time spent per question by group

Time spent per question			
	Control	Test	*P* value
Q1	22.72 ± 14.46	28.64 ± 12.86	.18
Q2	17.69 ± 19.71	15.49 ± 8.80	.66
Q3	4.44 ± 3.76	5.03 ± 5.81	.71
Q4	13.39 ± 19.06	8.88 ± 7.96	.34
Q5	33.36 ± 90.70	12.94 ± 15.68	.33

*Note*: Mean ± SD

Presence or absence CNN assistance did not significantly affect confidence level differences before and after the survey. The presurvey confidence between control and test groups was not statistically significant (Table 3) on a scale between 1 and 5, where 1 is equivalent to very low confidence and 5 is very high confidence (3.19 ± 0.85 and 3.25 ± 0.89, respectively). The postsurvey confidence between CNN assistance and no CNN assistance groups was closer to approaching statistical significance, yet not significant (3.29 ± 0.70 and 3.30 ± 0.78, respectively).

**Table 3.** Pre- and postsurvey confidence in ability to radiographically identify furcation involvement

	Presurvey confidence		Postsurvey confidence	
	Mean	*P* value	Mean	*P* value
Control	3.19 ± 0.85	.96	3.29 ± 0.70	.09
Test	3.25 ± 0.89		3.30 ± 0.78	
DS3	2.82 ± 0.83	.002*	2.95 ± 0.77	.007*
DS4	3.68 ± 0.65		3.68 ± 0.46	

*Note*. 1 indicates very low confidence and 5 indicates very high confidence.

Confidence levels were significantly different based on experience levels between third- and fourth-year dental students (Table 3). Prior to the survey third-year dental students were less confident than fourth-year dental students on their ability to radiographically identify furcation involvement (2.82 ± .083 and 3.68 ± 0.65, respectively; *P *=* *.002). After the survey third-year dental students were still less confident than fourth-year dental students on their ability to radiographically identify furcation involvement (2.95 ± 0.77 and 3.68 ± 0.46, respectively; *P *=* *.007).

Interactions with CNN-assisted radiographs of furcation involvement lesions did not significantly affect perceptions on helpfulness of a computer program to improve clinical confidence. Both control and test groups felt strongly that a computer program would help clinical confidence on furcation lesion identification on a scale from 1 to 5 with 1 denoting very low agreement and 5 as very high agreement (4.38 ± 0.58 and 4.05 ± 1.07, respectively). The control group responses ranged from 3 to 5 and the test group’s responses ranged from 1 to 5.

## DISCUSSION

AI models that provide clinical decision support to clinicians traditionally focus on accuracy of categorization. Despite improvements in data and AI models, many decision support systems fail in real-world clinical applications.^17^ The focus of this study was to investigate the AI-clinician interaction through accuracy, efficiency, and confidence of novice clinicians. Although a highly accurate decision support system might perform well without human interaction, we investigated the end-user accuracies that would ultimately be used to provide a diagnosis and treatment decision. These end-user accuracies could potentially impact clinical outcomes and/or patient harm particularly if misclassifications result in inappropriate treatment decisions. In our example, presence of radiographic FI is a factor that can decide periodontal surgical intervention.

### Accuracy

The accuracy of identifying FI in radiographs for inexperienced clinicians was generally not affected by AI assistance. However, in this study when presented with 1 tooth that posed a higher degree of diagnostic challenge, dental students were more likely to over-rely on AI assistance compared with those students without assistance. In the radiograph (Figure 1B, tooth on the right of the picture), the AI program had detected the presence of FI. Yet, the gold standard as labeled by 3 calibrated experienced periodontists was noted as no presence of FI. The inter-rater reliability between calibrated periodontists was 0.63 ± 0.05. In labeling FI, disagreements were solved by consensus. Radiographic identification of FI can be difficult to detect in some cases between experienced clinicians, therefore despite comparisons with a true gold standard, we found that when presented with AI assistance inexperienced clinicians are more likely to be biased by additionally presented information. Potential biases are anchoring, confirmation, automation, or complacency biases. This study did not attempt to discriminate effects of particular biases; however, this might be a potential future direction to attempt to minimize overall effect of biases.

### Efficiency

In this study, efficiency was measured based on completion time when presented with a diagnostic choice for individual and overall questions. Previous studies show that AI-assistance allows for faster radiographic identification for experienced clinicians.^8^^,^^9^ Although the findings of this study of inexperienced clinicians did not show statistically significant differences in overall speed of completion, the group with AI-assistance had much less variability and the magnitude of completion time favored AI assistance increasing speed of identification in general.

When presented with a more challenging diagnostic choice (Figure 1B, tooth on the right of the picture) and additional information (AI-assistance), a minimal level of experience potentially leads to increased time to diagnose using radiographs. In this study, it was found that participants with less experience in the AI-assistance condition performed slower to make a diagnostic decision. Some reasons for this effect might be due to presentation of too much visual information resulting in slower possibly less accurate decision-making.^18^ Although the results and design of the study limit deriving further conclusions.

One particular limitation of the study was that the survey was remotely administered. Therefore, the experimental environment was not standardized among subjects. Additionally, the number of questions on the survey was minimized to improve participation and completion of the survey, yet limited the sample size of diagnostic questions that were analyzed.

### Confidence

As expected, confidence levels were higher in the more experienced group. Despite the difference of 1-year clinical experience between groups, those in their fourth year of dental school were more confident in radiographic diagnosis of FI than those in their third year. Presence of AI-assistance did not affect confidence levels of students. Human trust is an important factor that can lead to confidence in AI to improve decision-making outcomes.^19^ In this study, participants were provided with text that noted the AI had a diagnostic accuracy of 80%. Therefore, the experimental conditions might have implicitly reduced trust in the AI and affected the outcomes of confidence levels.

Additionally, the presentation model of AI-assistance was in the form of graphical representation overlaid on the X-rays (Figure 1). This mode of presentation is therefore an explainable system versus the typical black-box decision of traditional AI models, which can improve confidence and thus acceptance in AI.^20^ The results of this study did not support that the presentation of AI in its current form changed confidence levels. It might be possible that the experience level of the clinician affects confidence in AI despite an explainable system. The environment was also limited to 1 survey without user feedback in a clinical setting that through continued use might build additional trust and confidence in the AI system. Additionally, the visual user interface (UI) might affect trust/confidence, and future studies to elucidate more beneficial presentation styles might be warranted.

### Perceptions of AI

Regardless of interaction with AI or lack of interaction with AI participants in this study generally felt strongly that software can improve clinical confidence in identifying FI. Dental students are exposed to multiple digital technologies that already use AI. Although most might not be aware of the AI within the technologies, the students surveyed are willing to accept help from an AI-based system.

The accuracy, efficiency, and confidence measured in novice clinicians provide insight into the human–AI interactions in a clinical detection task. However, limitations exist in this single study with limited number of participants. No attempt was made to identify or discriminate biases. The survey was remotely administered and thus the experimental environment was not standardized. Additionally, a limited amount of diagnostic questions were asked of participants. A larger study with a standardized environment testing a different clinical task would improve the generalizability of the results found in this study. Future directions might also attempt to discern if interactions are affected by provider knowledge of varying degrees of AI accuracy and its effect of human–AI trust. In addition, these findings were in the context of novice clinicians and it is possible that experienced clinicians might not show the same effects.

## CONCLUSION

Though the integration of AI into healthcare has great potential, caution must be taken with its use. Given the experience level of a clinician and a more challenging diagnostic choice, presenting additional information will likely decrease the efficiency and increase potential for biases. Diagnostic inaccuracies can lead to incorrect treatment as well as delay correct diagnosis and treatment, which can have serious consequences for patients.^21^ When implementing AI systems for diagnostic decision-making, not only is the visual presentation of the UI important but the psychological environment associated with the AI-clinician relationship is important to consider as well.

## FUNDING

This work was supported by the University of Texas Health Science Center School of Dentistry grant programs: (1) Student Research Program and (2) Dean’s Academy Small Grants Program.

## AUTHOR CONTRIBUTIONS

All authors meet the criteria for authorship based on the ICMJE guidelines.
